# Approximate Solutions for Flow with a Stretching Boundary due to Partial Slip

**DOI:** 10.1155/2014/747098

**Published:** 2014-11-24

**Authors:** U. Filobello-Nino, H. Vazquez-Leal, A. Sarmiento-Reyes, B. Benhammouda, V. M. Jimenez-Fernandez, D. Pereyra-Diaz, A. Perez-Sesma, J. Cervantes-Perez, J. Huerta-Chua, J. Sanchez-Orea, A. D. Contreras-Hernandez

**Affiliations:** ^1^Electronic Instrumentation and Atmospheric Sciences School, University of Veracruz, Circuito Gonzalo Aguirre Beltrán S/N, 91000 Xalapa, VER, Mexico; ^2^National Institute for Astrophysics, Optics and Electronics, Luis Enrique Erro No. 1, Santa Maria Tonantzintla, 72840 Puebla, PUE, Mexico; ^3^Abu Dhabi Men's College, Higher Colleges of Technology, P.O. Box 25035, Abu Dhabi, UAE; ^4^Civil Engineering School, University of Veracruz, Venustiano Carranza S/N, Colonia Revolucion, 93390 Poza Rica, VER, Mexico

## Abstract

The homotopy perturbation method (HPM) is coupled with versions of Laplace-Padé and Padé methods to provide an approximate solution to the nonlinear differential equation that describes the behaviour of a flow with a stretching flat boundary due to partial slip. Comparing results between approximate and numerical solutions, we concluded that our results are capable of providing an accurate solution and are extremely efficient.

## 1. Introduction

According to the classification of Prandtl, the fluid motion is divided into two regions. The first region is near the object where the effect of friction is important and is known as the boundary layer, while, for the second type, these effects can be neglected [1–3]. It is common to define the boundary layer as the region where the fluid velocity parallel to the surface is less than 99% of the free stream velocity [1].

The boundary layer thickness *δ* increases from the edge along the surface on which the fluid moves. Even for the case of a laminar flow, the exact solution of equations describing the laminar boundary layer is very difficult to calculate and only few simple problems can straightforward be analysed [1, 3].

**Figure 1:**
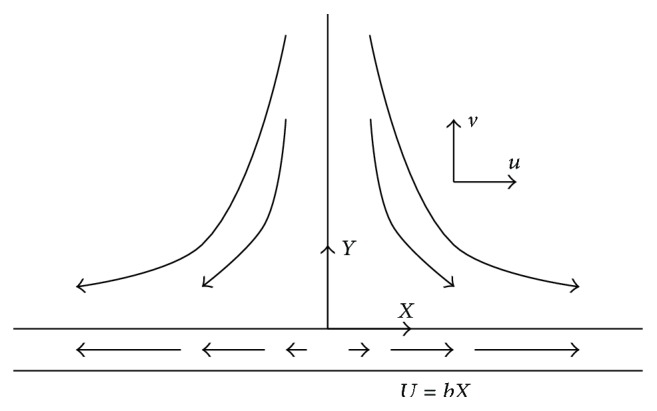
Schematic showing a stretching boundary.

An interesting case is the one where a flow is induced into a viscoelastic fluid by a linearly stretched sheet [4–6] (see Figure 1). Extrusion of molten polymers through a slit die for the production of plastic sheets is an important process in polymer industry [4]. The process is normally complicated from the physical point of view, because it requires significant heat transfer between the sheet and a surrounding fluid that plays the role of a cooling medium. An important aspect of the flow is the extensibility of the sheet which can be employed to improve its mechanical properties along the sheet. To obtain better results is necessary to improve the cooling rate, whereby it is common to add some polymeric additives into water (which is one of the most employed fluids as cooling medium) in order to have a better control on the cooling rate. The flow due to a stretching boundary is also important in other engineering processes of interest, such as the glass fibre drawing and crystal growing among many others. Detail discussion about this topic can be found in [4]. This work assumes that the boundary conditions for the problem under study are adequately described by Navier's condition, which states that the amount of relative slip is proportional to local shear stress. Unlike what happens with fluids like water, mercury, and glycerine, which do not require slip boundary conditions [7], there are cases where partial slip between the fluid and the moving surface may occur. Some known cases include emulsions, as mustard and paints and polymer solutions and clay [7].

He [8, 9] proposed the standard HPM; it was introduced as a powerful tool to approach several kinds of nonlinear problems. The HPM can be considered as a combination between the classical perturbation technique and the homotopy (whose origin is in the topology), but not restricted to the limitations found in traditional perturbation methods. For instance, HPM method does need neither small parameter nor linearisation, just few iterations to obtain accurate results [5, 8–35]. The fundamentals of HPM convergence can be found in [21, 24, 25].

There are other modern alternatives to find approximate solutions to the differential equations that describe some nonlinear problems such as those based on variational approaches [36–39], tanh method [40], exp-function [41, 42], Adomian decomposition method [43–49], parameter expansion [50], homotopy analysis method [4, 51, 52], and perturbation method [53] among many others.

To figure out how HPM method works, consider a general nonlinear equation in the form

(1) $$\begin{array}{c} A\left(u\right)-f\left(r\right)=0,\;r\in \Omega , \end{array}$$

with the following boundary conditions:

(2) $$\begin{array}{c} B\left(u,\frac{\partial u}{\partial n}\right)=0,\;r\in \Gamma , \end{array}$$

where *A* is a general differential operator, *B* is a boundary operator, *f*(*r*) is a known analytical function, and Γ is the domain boundary for *Ω*.

Also, *A* can be divided into two parts *L* and *N*, where *L* is linear and *N* nonlinear; from this last statement, (1) can be rewritten as

(3) $$\begin{array}{c} L\left(u\right)+N\left(u\right)-f\left(r\right)=0. \end{array}$$

In a broad sense, a homotopy can be constructed in the following form [8, 9]:

(4) Hv,p=1−pLv−Lu0 +pLv+Nv−fr=0,WWWWinWp∈0,1, r∈Ω,

or

(5) Hv,p=Lv−Lu0+pLu0+Nv−fr=0,p∈0,1, r∈Ω,

where *p* is a homotopy parameter, whose values are within range of 0 and 1, and *u*_0_ is the first approximation to the solution of (3) that satisfies the boundary conditions. Assuming that solution for (4) or (5) can be written as a power series of *p*

(6) $$\begin{array}{c} v=v_{0}+v_{1}p_{1}+v_{2}p_{2}^{2}+\cdots . \end{array}$$

Substituting (6) into (5) and equating identical powers for *p* terms, it is possible to obtain the values for the sequence *u*_0_, *u*_1_, *u*_2_,….

When *p* → 1, it yields in the approximate solution for (1) in the form

(7) $$\begin{array}{c} v=v_{0}+v_{1}+v_{2}+v_{3}+\cdots . \end{array}$$

Another way to build a homotopy, which is relevant for this paper, is by considering the following general equation:

(8) $$\begin{array}{c} L\left(v\right)+N\left(v\right)=0, \end{array}$$

where *L*(*v*) and *N*(*v*) are the linear and nonlinear operators, respectively. It is desired that solution for *L*(*v*) = 0 describes, accurately, the original nonlinear system.

By the homotopy technique, a homotopy is constructed as follows [18]:

(9) $$\begin{array}{c} \left(1-p\right)L\left(v\right)+p\left[L\left(v\right)+N\left(v\right)\right]=0. \end{array}$$

Again, it is assumed that solution for (9) can be written in the form (6); thus, taking the limit when *p* → 1 results in the approximate solution for (8).

The variation of homotopic parameter within the range [0,1] amounts to a deformation that begins from an initial equation with known solution until it becomes the equation to be solved. From a practical point of view, taking the limit *p* → 1 is just setting *p* = 1.

## 2. Padé Approximant

Let *u*(*t*) be an analytical function with the Maclaurin's expansion

(10) $$\begin{array}{c} u\left(t\right)=\overset{\infty }{\underset{n=0}{\sum }}u_{n}t^{n},\;0\leq t\leq T. \end{array}$$

Then the Padé approximant to *u*(*t*) of order [*L*, *M*] which we denote by [*L*/*M*]_*u*_(*t*) is defined by [54–57]

(11) $$\begin{array}{c} {\left[\frac{L}{M}\right]}_{u}\left(t\right)=\frac{p_{0}+p_{1}t+\cdots +p_{L}t^{L}}{1+q_{1}t+\cdots +q_{M}t^{M}}, \end{array}$$

where we considered *q*_0_ = 1, and the numerator and denominator have no common factors.

The numerator and the denominator in (11) are constructed so that *u*(*t*) and [*L*/*M*]_*u*_(*t*) and their derivatives agree at *t* = 0 up to *L* + *M*. That is,

(12) $$\begin{array}{c} u\left(t\right)-{\left[\frac{L}{M}\right]}_{u}\left(t\right)=O\left(t^{L+M+1}\right). \end{array}$$

From (12), we have

(13) $$\begin{array}{c} u\left(t\right)\overset{M}{\underset{n=0}{\sum }}q_{n}t^{n}-\overset{L}{\underset{n=0}{\sum }}p_{n}t^{n}=O\left(t^{L+M+1}\right). \end{array}$$

From (13), we get the following algebraic linear systems:

(14) uLq1+⋯+uL−M+1qM=−uL+1uL+1q1+⋯+uL−M+2qM=−uL+2⋮uL+M−1q1+⋯+uLqM=−uL+M,

(15) p0=u0p1=u1+u0q1⋮pL=uL+uL−1q1+⋯+u0qL.

From (14), we calculate first all the coefficients *q*_*n*_, 1 ≤ *n* ≤ *M*. Then, we determine the coefficients *p*_*n*_, 0 ≤ *n* ≤ *L* from (15).

Note that for a fixed value of *L* + *M* + 1, the error (12) is the smallest when the numerator and denominator of (11) have the same degree or when the numerator has one degree higher than the denominator.

## 3. Laplace-Padé Resummation Method

Several approximate methods provide power series solutions (polynomial). Nevertheless, sometimes, this type of solutions lacks large domains of convergence. Therefore, Laplace-Padé resummation method [54] is used in literature to enlarge the domain of convergence of solutions or inclusive to find exact solutions.

The Laplace-Padé method can be explained as follows.

(1) First, Laplace transform is applied to power series.
(2) Next, *s* is substituted by 1/*t* in the resulting equation.
(3) After that, we convert the transformed series into a meromorphic function by forming its Padé approximant of order [*N*/*M*]. *N* and *M* are arbitrarily chosen, but they should be of smaller values than the order of the power series. In this step, the Padé approximant extends the domain of the truncated series solution to obtain better accuracy and convergence.
(4) Then, *t* is substituted by 1/*s*.
(5) Finally, by using the inverse Laplace *s* transform, we obtain the exact or approximate solution.

## 4. Formulation

Consider a two-dimensional stretching boundary (see Figure 1). Experiments show that the velocity of the boundary *U* is approximately proportional to the distance from the orifice *X* [7, 58], so that

(16) $$\begin{array}{c} U=bX, \end{array}$$

where *b* is a proportionality constant.

Let (*u*, *v*) be the fluid velocities for the (*X*, *Y*) directions, respectively. In this case the boundary condition is adequately described by Navier's condition, which states that the amount of relative slip is proportional to local shear stress:

(17) $$\begin{array}{c} u\left(X,0\right)-U=k\nu \frac{\partial u}{\partial Y}\left(X,0\right), \end{array}$$

where *k* is a constant of proportionality and *ν* is the kinematic viscosity of the bulk fluid. The relevant expressions for this case are the Navier-Stokes equations:

(18) uuX+vuY+pXρ−νuXX+uYY0,

(19) uvX+vvY+pYρ−νvXX+vYY=0,

and continuity

(20) $$\begin{array}{c} u_{X}+v_{Y}=0, \end{array}$$

where *ρ* and *p* are density and pressure, respectively.

To solve equations (18)–(20), we have to consider boundary conditions (16) and (17), besides the fact that there is no lateral velocity or pressure gradient away from the stretching surface.

Next, we will show that it is possible to get an ordinary nonlinear differential equation from (18).

With this end, we note that *Y* component of velocity is negative (*v* < 0) and by symmetry arguments it only depends on *Y* (see Figure 1). Therefore, it is possible to define a function *y*(*x*) ≥ 0, 0 ≤ *x* < *∞*, such that [7]:

(21) $$\begin{array}{c} v=-\sqrt{b\nu }y\left(x\right), \end{array}$$

where

(22) $$\begin{array}{c} x=Y\sqrt{\frac{a}{\nu }}. \end{array}$$

From (21) and continuity equation (20) we obtain

(23) $$\begin{array}{c} u=bXy^{\prime }\left(x\right). \end{array}$$

Clearly (20) is satisfied under transformations (21), (22), and (23), while (18) adopts the simpler form;

(24) $$\begin{array}{c} y^{\prime \prime \prime }\left(x\right)-{\left(y^{\prime }\left(x\right)\right)}^{2}+y\left(x\right)y^{\prime \prime }\left(x\right)=0. \end{array}$$

To deduce the boundary conditions of (24), we see from Figure 1 that *v*(*Y* = 0) = 0 and lim⁡_*Y*→*∞*_⁡*u*(*X*, *Y*) = 0 and therefore

(25) y00,y′∞=0.

Finally, substituting (16) and (23) into (17) we obtain

(26) $$\begin{array}{c} y^{\prime }\left(0\right)=k\sqrt{b\nu }y^{\prime \prime }\left(0\right)+1. \end{array}$$

## 5. Approximate Solution for a Two-Dimensional Viscous Flow Equation

Next, we present some solution methods to the study problem.

### 5.1. HPM Method

In this section, HPM is used to find approximate solutions for (24). Identifying the linear part as

(27) $$\begin{array}{c} L=y^{\prime \prime \prime }, \end{array}$$

and the nonlinear as

(28) $$\begin{array}{c} N=-{\left(y^{\prime }\right)}^{2}+yy^{\prime \prime }, \end{array}$$

we initiate the HPM method by constructing a homotopy based on (9), in the form

(29) $$\begin{array}{c} \left(1-p\right)y^{\prime \prime \prime }+p\left(y^{\prime \prime \prime }-{\left(y^{\prime }\right)}^{2}+yy^{\prime \prime }\right)=0. \end{array}$$

Assuming that the solution has the form [8, 9]:

(30) $$\begin{array}{c} y=y_{0}+y_{1}p_{1}+y_{2}p_{2}^{2}+\cdots \text{.} \end{array}$$

Then, substituting (30) into (29) and equating terms having identical powers of *p* we obtain

(31) p0:  y0′′′=0,p1:  y1′′′−y0′2+y0y0″=0,p2:  y1y0″−2y0′y1′+y2′′′+y0y1″=0,p3:  y2″+y1y1″−y1′2+y2y0″+y3′′′−2y0′y2′=0,p4:  y1″+y0y3″+y1y2″−2y0′y3′+y3y0″+y4′′′−2y1′y2′=0,p5:  y3″−y2′2+y5′′′−2y1′y3′+y4y0″+y2y2″−2y0′y4′+y0y4″+y3y1″=0,p6:  y5y0″+y0y5″+y6′′′−2y0′y5′+y4y1″+y1y4″+y2y3″−2y1′y4′+y3y2″−2y2′y3′=0,p7:  y4″−y3′2+y4y2″+y1y5″+y0y6″+y6y0″−2y2′y4′+y5y1″+y3y3″−2y0′y6′+y7′′′−2y1′y5′=0.

In order to fulfil the boundary conditions from (24) given by (25)-(26), we find that *y*_0_(0) = 0, $y_{0}^{\prime }(0)=1+k\sqrt{b\nu }a$ , *y*_0_^″^(0) = *a*, *y*_1_(0) = 0, *y*_1_′(0) = 0, *y*_1_^″^(0) = 0, *y*_2_(0) = 0, *y*_2_′(0) = 0, *y*_2_^″^(0) = 0, *y*_3_(0) = 0, *y*_3_′(0) = 0, *y*_3_^″^(0) = 0, and so on. We have assumed the value for *y* ^″^(0) as some adequate constant *a*, which adopts the following values for the corresponding *k* as follows: {*k* = 0, *a* = −1}, {*k* = 0.3, *a* = −0.701}, {*k* = 1, *a* = −0.430}, {*k* = 2, *a* = −0.284}, {*k* = 5, *a* = −0.145}, and {*k* = 20, *a* = −0.0438} [6]. Thus, the results obtained from above equations are

(32) y0=x1+12x+ka,y1=161+14xk+120x2+k2a2+2k+14xax3,y2=ax67201+−17xk−156x2+k2a2WWiWiW+2k−17xa,y3=x725201+18xk3+3160x3kWWWWiWWW+116x2k2+31760x4+k4a4WWiiWnW+3160x3+4k3+18x2k+38xk2a3WWiiWnW+6k2+116x2+38xka2+4k+18xa,y4=−x9453601+1340xk4+29440x2k3+317640640x5WWWWWWnWW+1013520x3k2+31745760x4k+k5a5WWWWWW+87440x2k2+5k4+1310xk3WWWWWWWn+31745760x4+1011760x3ka4WWWWWW+87440x2k+3920xk2+1013520x3+10k3a3WW−x94536010k2+29440x2+1310xka2WWWWWW+5k+1340xa,y5=112474001+58917472x3k3+37208x2k4WWWnWWWWW+41926880x4k2+1924xk5WWWWWWnWW+343119009536x6+k6+34311118208kx5a6WWWiWWW+3752x2k3+6k5+5895824x3k2+34311118208x5WWWWWWWW+9524xk4+41913440x4ka5x11WW+11247400111104x2k2+15k4+41926880x4WWWWWWWW+5895824x3k+9512xk3a4WWWWWnWW+20k3+9512xk2+3752x2k+58917472x3a3WWWWnWWW+9524xk+37208x2+15k2a2WWWWnWWW+1924x+6kax11,y6=1972972001+−59960928x4k3−351224xk6−169326880x3k4WWWWWnWWWW−32573360x2k5+k7−351372437120x5k2WWWWWWnWWW−12429146305280x6x−124291926105600x7a7WWWWWWW+−3257672x2k4−16936720x3k3−351371218560x5kWWWWWWWWW−1053112xk5−12429146305280x6WWWWWWWWW+7k6−179760928x4k2x13WW+197297200WW×a6+−3257336x2k3+21k5−16934480x3k2WWWWWWW−351372437120x5−179760928x4k−5265224xk4a5WWWW+−3257336x2k2+35k4−59960928x4WWWWWW−16936720x3k−175556xk3a4x13WW+197297200WW×35k3−5265224xk2−3257672x2k−169326880x3a3WWW+−1053112xk+21k2−32573360x2a4x13,y7=−239081072000WW×1+k8−1875011808351272960x8+395552xk7WWWWWW−187501135145707520x7k−3923034792596480x6k2WWWWWW+27080568465664x5k3+103791751349248x4k4WWWWWW+50751351296x3k5+8393225216x2k6a8x15WW−239081072000WW×1037917808351272960x4k3+839337536x2k5+8k7WWWW+2765552xk6+253751351296x3k4−187501135145707520x7WWWW+27080522821888x5k2−3923032396298240x6ka7x15WW−239081072000WW×25375675648x3k3+10379178558208x4k2WWWW+27080522821888x5k+2765184xk5WWWW+4196575072x2k4−3923034792596480x6+28k6a6x15WW−239081072000WW×4196556304x2k3+56k5+25375675648x3k2WWWW+27080568465664x5+13825552xk4WWWW+103791712837312x4k+4196575072ax2k2a5x15WW−239081072000WW×70k4+103791751349248x4+253751351296x3k5075x31351296aWWWW+13825552xk3+56k3a+2765xk2184aWWWW+8393x2k37536a+5075x31351296a+2765xk552a2a4x15WW−2390810720008393225216x6+28k2a2x15WW−239081072000395x552+8kax15,

and so on.

To exemplify, we will consider the seventh order approximation by substituting solutions (32) into (30) and calculating the limit when *p* → 1:

(33) $$\begin{array}{c} y=\underset{p\to 1}{\lim}\left(\overset{7}{\underset{i=0}{\sum }}y_{i}p^{i}\right). \end{array}$$

The above approximation is valid for all values of *k* ≥ 0 and corresponds to the results reported in [5] for the cases when *k* = 0 and *k* = 20. Clearly, if higher order approximations are considered, better accuracy is obtained but the resulting expressions could be too long.

### 5.2. HPM Laplace-Padé Scheme (LPHPM)

Next, we study the case *k* = 0; it means that *y*′(0) = 1 (see (26)). This is an interesting case because the following exact solution was reported in [59]:

(34) $$\begin{array}{c} y\left(x\right)=1-\exp\left(-x\right). \end{array}$$

Thus, substituting *k* = 0 in (33) we obtain the following series solution:

(35) yx−0.5x2+x−7.878129877×10−11x19+1.13972148927×10−11x20+1.57691357363×10−10x18+2.07320134446×10−13x21−1.35120811977×10−13x22+5.87481791204×10−15x23+0.16666667x3−0.041666666x4+0.00833333333x5−0.00138888888x6+0.000198412698x7−0.0000248015873x8−2.755731×10−7x10+0.000002755x9+2.5052108×10−8x11−2.087675×10−9x12+1.605904×10−10x13−1.147074559×10−11x14+7.647163731×10−13x15−4.779477332×10−14x16−5.073836906×10−11x17.

As we will see (35) is accurate only for small values of *x*. To guarantee the validity of the approximate solution (35) for large values of this variable, the series solution is transformed using the Padé approximation and Laplace transform (see Section 3). As first step, Laplace transform [54] is applied to (35):

(36) −1s3+1s2−9.583359×106s20+2.77283070001×107s21+1.009599×106s19+1.0592181×107s22−1.51875891×108s23+1.51875891×108s24+1s4−1s5+1s6−1s7+1s8−1s9−1s11+1s10+0.9999999998s12−1s13+0.9999999999s14−0.9999999997s15+1s16−1s17−18047s18,

and then, *s* is substituted by 1/*x* in the equation to obtain

(37) −x3+x2−9.583359×106x20+2.77283070001×107x21+1.009599×106x19+1.0592181×107x22−1.51875891×108x23+x4−x5+x6−x7+x8−x9−x11+x10+0.9999999998x12−x13+0.9999999999x14−0.9999999997x15+x16−x17−18047x18+1.51875891×108x24.

Following Laplace-Padé scheme, Padé approximant [6/6] is applied to obtain

(38) $$\begin{array}{c} \frac{x^{2}}{1+x}; \end{array}$$

here *x* is substituted by 1/*s*; the result is

(39) $$\begin{array}{c} \frac{1}{s\left(s+1\right)}. \end{array}$$

Finally, by means of the inverse Laplace transform applied to (39), we obtain the exact solution (34) for (24).

### 5.3. HPM Padé Scheme (PHPM)

Another way to recover lost information from the truncated series (33) is by means of applying the Padé approximant [7/7]. For the particular case when *k* = 0 (see (35)), we obtain the following rational approximation for (24) (using dummy variables *a*1 and *b*1)

(40) a11.15625115625×10−7x7+0.000174825174825x5+0.0320512820513x3+x,b11+0.1153846153x2+0.001456876456x4+0.5x+0.01602564102x3+0.00008741258741x5+0.00000323750323x6+5.781255781×10−8x7,

and therefore, the final result is

(41) $$\begin{array}{c} y\left(x\right)=\frac{a1}{b1}. \end{array}$$

## 6. Discussion

**Figure 2:**
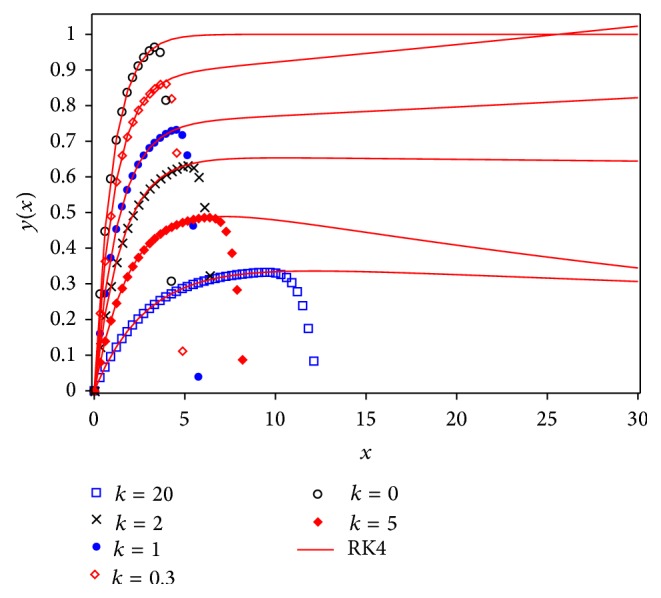
Fourth order Runge Kutta numerical solution for (24) (solid line) and HPM solution (33) (symbols).

**Figure 3:**
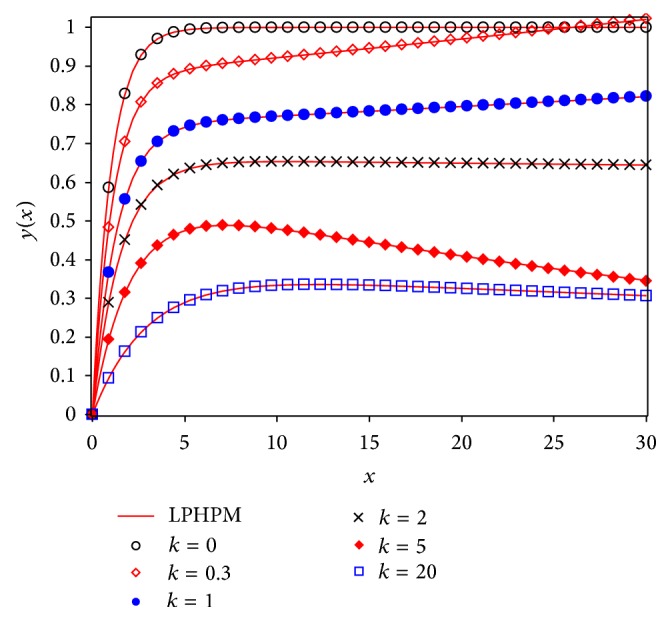
Fourth order Runge Kutta solution for (24) (symbols) and LPHPM (solid line).

**Figure 4:**
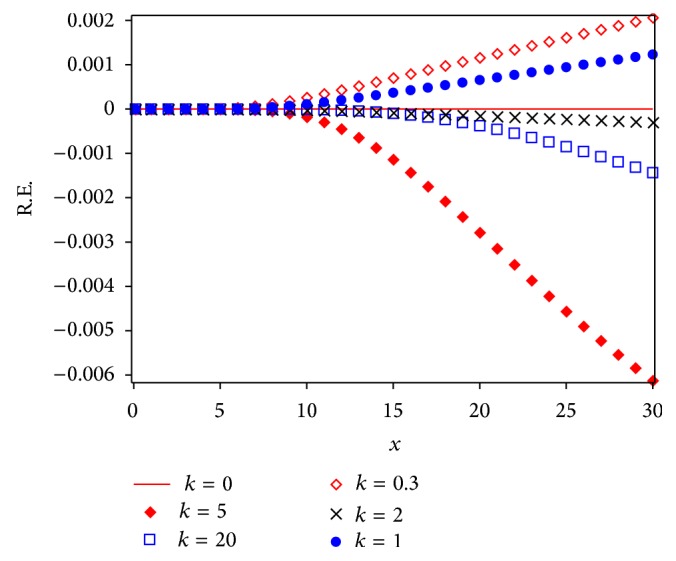
Relative error for different cases of LPHPM.

**Figure 5:**
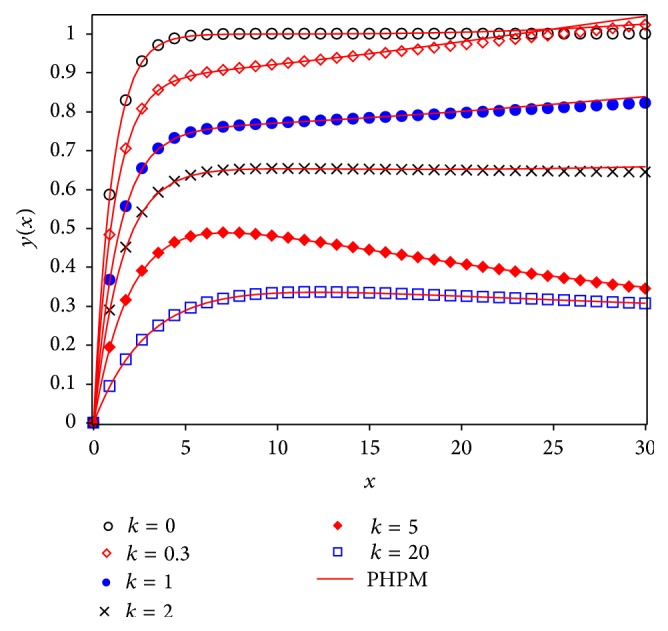
Fourth order Runge Kutta numerical solution for (24) (symbols) and PHPM (solid line).

**Figure 6:**
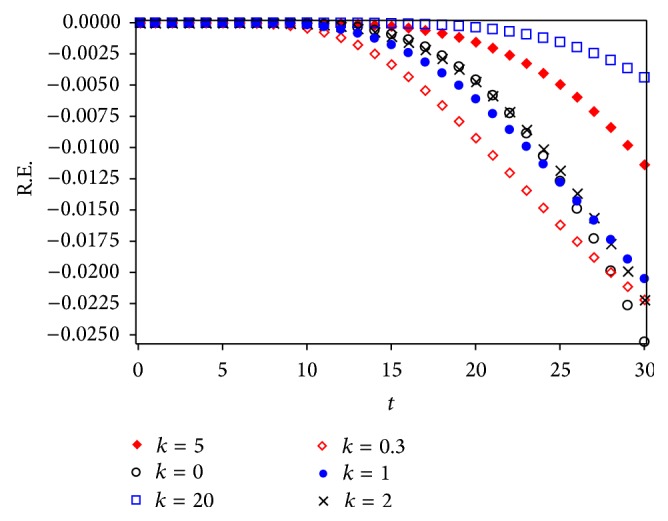
Relative error for different cases of PHPM.

Figures 3 and 5 show a comparison between Runge Kutta 4 (RK4) numerical solution for different values of *k* and approximations given by Laplace-Padé and Padé methods applied to (33). From Figures 4 and 6 it can be noticed that the relative error obtained by our approximations was low. As a matter of fact, the largest error for LPHPM is −0.006 when *k* = 5 and for PHPM is −0.0250 when *k* = 0. In a broad sense, if higher order approximations are considered, higher accuracy is obtained from LP-HPM and PHPM methods. In particular, it is noteworthy that Laplace-Padé method allows recovering the exact solution (34) when *k* = 0 from the truncated series (33). This contrasts with Figure 2, which shows the comparison between RK4 and HPM approximations given by (33) for *k* = 0,0.3,1, 2,5 and *k* = 20. It is evident that HPM series is accurate only for a restricted domain of values for the independent variable *x*. Also it is worth comparing the cumbersome approximation (35) for *k* = 0, with the handy expressions (34) and (41) obtained by LHPM and PHPM, respectively. In [5] HPM was employed to solve (24) with a good approximation for a restricted domain of values for *x* and small values for *k*, while in [6] the same equation was solved using perturbation method (PM) for small values of *k*. Indeed it is known that PM provides, in general, better results for small values of the perturbation parameter. Unlike the LPHPM and PHPM schemes were employed to obtain accurate solutions for different values of *k*, having low relative errors for *x* values within the range 0 ≤ *x* ≤ 30, as shown in Figures 4 and 6.

The importance of providing analytical solutions, although approximate, with good accuracy is that numerical solutions only provide a qualitative idea of the problem to be solved. Besides, just as it was shown in one of our case studies, a solution obtained by a numerical method like Runge Kutta could hide the case of an exact solution. This possibility was successfully explored by LPHPM for case study *k* = 0. Finally, another reason why we are interested in obtaining analytical approximation solutions is that numerical algorithms could give some problems, such as numerical instabilities and oscillations, among others. This means that the numerical solutions may not correspond to the real solution of the original differential equation [60].

Just as it was seen with our HPM solutions, one disadvantage of approximating them with polynomials is its tendency to oscillate; this gives rise to the fact that the obtained solutions diverge, especially for the case of problems defined on open intervals. This can be attributed to the possibility that the radius of convergence may not be sufficiently large to contain the boundaries of the domain of study [56, 61].

In order to improve the aforementioned, the use of rational functions was proposed, through the Padé approximation method.

The Padé approximation is an extension of the Taylor polynomial approximation but for rational functions. When the denominator of the approximation is a zero degree polynomial function, the Padé approximation is reduced to a Maclaurin polynomial.

Padé-approximate extrapolation technique consists in approximating a truncated series (such as those resulting of HPM) by a rational function; the latter extends the range of validity of the initial polynomial. The above results are particularly relevant if the truncated series represent the solution of a differential equation.

We observe that even though the series has a finite region of convergence, Padé approximant allows obtaining the limit of the function under study as *x* takes large values, if *L* = *M* (see Figures 3 and 5) [55]. In fact, the rational functions whose denominator and numerator have the same values of *L* and *M*, or a degree almost identical, give rise to results better than those obtained by methods based on polynomial functions [61].

Finally, the convergence is uniform in any compact region in the case of Padé approximation, whereas truncated Maclaurin series is valid only in a near neighbourhood of zero point. The above explains why HPM-Padé has larger intervals of convergence in comparison with HPM series [55].

As it was already mentioned, LPHPM applies Padé approximant to the resulting expression, derived of applying Laplace transform to the HPM truncated series, and the rational function obtained in this way acquires the benefits of the abovementioned Padé method.

Nevertheless, one possible advantage of LPHPM is that the application of inverse Laplace transform at the last step of the method could result in the exact solution of the problem.

## 7. Conclusions

This work showed that some nonlinear problems may be adequately approximated using the coupling of the HPM method with Laplace-Padé and Padé methods to deal with HPM truncated power series. For instance, the flow induced by a stretching sheet is adequately described by our approximations given by LPHPM and PHPM (see Figure 3 through Figure 6). Figures 4 and 6 illustrate the relative error and show that the proposed solutions are highly accurate. Since that this procedure is, in principle, applicable to other similar problems, we conclude that LPHPM and PHPM are methods with high potential in the search for analytical approximate solutions for nonlinear problems.
